# The Impact of Negative Workplace Gossip on Employees’ Organizational Self-Esteem in a Differential Atmosphere

**DOI:** 10.3389/fpsyg.2022.854520

**Published:** 2022-06-17

**Authors:** Xiaolei Song, Siliang Guo

**Affiliations:** ^1^Student Affairs Department, Qilu Normal University, Jinan, China; ^2^Graduate School Education Management Department, Nueva Ecija University of Science and Technology, Cabanatuan City, Philippines; ^3^School of Economics and Management, Qilu Normal University, Jinan, China

**Keywords:** workplace negative gossip, workplace exclusion, poor-order atmosphere, organizational self-esteem, intermediary effect

## Abstract

The level of organizational self-esteem of employees, whether on the production line or as managers or directors of enterprises, does not only correlate with individual performance but has also become a key factor in determining the completion of team core tasks. Based on the theory of self-consistency, this study explores the correlation between negative workplace gossip and employees’ organizational self-esteem by revealing the intermediary role of workplace exclusion and poor-order atmosphere. A survey of 228 employees from enterprises in Shandong and Shanghai showed that negative workplace gossip exerted a significant negative impact on employees’ organizational self-esteem, suggesting that negative workplace gossip reduces employees’ organizational self-esteem in the context of Chinese organizations. In addition, workplace exclusion exerted a complete intermediary effect between negative workplace gossip and employees’ organizational self-esteem, and poor-order atmosphere perception played a partial intermediary role. This study uncovers the black box that negative workplace gossip affects employees’ organizational self-esteem and has a strong enlightening significance for management practice.

## Introduction

In the new era, the objective of employees to enter the workplace is no longer simply to fulfill material needs but also to attain self-worth to attain spiritual satisfaction. In the complex and changeable market environment, employees’ performance has always been a topic of concern in management and economics. Organizational self-esteem has a significant impact on employee performance (Pierce et al., 1989). Employees with different levels of organizational self-esteem exhibit different performance in the enterprise. Employees with high-level organizational self-esteem are typically opinion leaders in the organization, and they have a strong sense of responsibility (Pierce et al., 1989). Usually, they come forward to exhibit their abilities at critical moments. When organizations face difficulties, they empathize with each other and have a strong sense of ownership and mission. Contrarily, employees with a low-level organizational self-esteem lack confidence in their ability and value and often have a low sense of organizational identity; thus, it is challenging to mobilize their enthusiasm for work and create better value for the enterprise.

Previous research mainly focused on the intermediary effect of organizational self-esteem (Yang et al., 2016; Li and Tian, 2017) and the impact of organizational self-esteem on employees’ attitude and behavior (Korman, 1970; Ghosh et al., 2012; Decoster et al., 2013; Gu and Yu, 2020), and few studies (Lu and Tu, 2014; Ahmed et al., 2021) focused on variables that affect organizational self-esteem. The research on the antecedent variables of organizational self-esteem primarily starts from two aspects: individual characteristics and organizational characteristics. For example, overall self-esteem, self-efficacy, need for success, organizational setting, organizational fairness, organizational ownership (Ferris et al., 2009), and the quality of the exchange relationship between leaders and subordinates positively correlate with organizational self-esteem (Kong et al., 2020). Conversely, negative emotion, gender discrimination, and negative workplace gossip negatively correlate with organizational self-esteem (Ellwardt et al., 2012). Negative workplace gossip is common in many workplaces (Foster, 2004; Samnani and Singh, 2016; Du et al., 2017), however, as a negative workplace phenomenon, it might bring many adverse effects to enterprises and employees (Ming, 2018). Negative workplace gossip has three crucial characteristics: (i) it spreads quickly and is accompanied by a magnifying effect (Yue et al., 2015); (ii) the degree of its influence depends largely on the subjective perception of the person being gossiped (Persson et al., 2016); and (iii) negative workplace gossip is hidden, which makes it extremely challenging to trace the source of gossip (Beersma and Kleef, 2012). Some studies (Pierce et al., 1989; Samnani et al., 2014) proposed that negative workplace gossip often damages the emotional relationship between employees through the spread of negative information, which results in the occurrence of workplace exclusion. Conversely, individuals experiencing workplace exclusion decrease their psychological cognition and assessment of the organization because of the rupture of interpersonal relationships (Williams et al., 2005). In addition, some studies (Yue et al., 2015; Du and Zhu, 2018) reported that in the Chinese organizational context, a close relationship exists between employees’ organizational self-esteem and the atmosphere of team order. As a negative organizational atmosphere, in the environment of a strong poor order, employees’ perceived unfair treatment due to negative workplace gossip does not match their fair and just values, which is highly likely to cause employees’ cognitive dissonance, which might lead to further reduction of employees’ organizational self-esteem. Unfortunately, currently, the research on the impact of workplace negative gossip on employees’ organizational self-esteem is still in its infancy (Du et al., 2017; Liu and Zong, 2019), and the correlation between negative gossip and many intermediary variables still lacks theoretical analysis and empirical test (Gkorezis et al., 2016; Du and Zhu, 2020). Although some studies (Jose et al., 2015; Peng et al., 2016) have explored negative workplace gossip, not much attention has been paid to the mechanism and boundary conditions of how it affects employees’ organizational self-esteem.

Self-consistency theory can provide an explanation for the relationship between workplace negative gossip and organizational self-esteem. The self-consistency theory was proposed by Aronson and Golden (1962) and held that a person’s motivation was consistent with his sense of self-worth, and a person with a high sense of self-worth was mainly governed by self-enhancement motivation. According to the self-consistency theory, individuals strive to keep their self-cognition consistent with the expectations and comments of others, and their behavioral responses will be consistent with their self-cognition (Wu et al., 2020). Negative workplace gossip refers to the spread of negative comments or negative information that individuals perceive others to have made about them in the workplace. Negative workplace gossip can bring negative self-perception to the person being gossiped about. Such negative self-perception will make the employees who are gossiped about have a strong sense of rejection in the workplace, meanwhile, will make them more sensitive to the internal atmosphere of difference in the organization. Both of these two aspects have a negative impact on the perception of value and importance in the organization of employees who are gossiped about, resulting in a lower sense of organizational self-esteem. Overall, considering the significance of employees’ organizational self-esteem to the whole organization or team, this study is based on the self-consistency theory and constructs a multi-intermediary model in which negative workplace gossip affects organizational self-esteem. Specifically, the model discusses how negative workplace gossip affects employees’ organizational self-esteem, as well as the mediating effects of workplace exclusion and poor-order atmosphere perception. Different from previous studies, this study incorporated workplace negative gossip and employee organizational self-esteem into a unified research framework, and explored the influence relationship and internal mechanism. The contribution of this paper are as follows: (1) Empirically tests the effect of negative gossip on employees’ organizational self-esteem, which enriches the research content of organizational self-esteem. (2) Analyzes the impact path of negative gossip on employees’ organizational self-esteem, and further clarify the functional mechanism and boundary conditions of negative gossip on employees’ organizational self-esteem. (3) Introduces poor-order atmosphere, a variable of organizational environment influenced by Chinese culture, to empirically test the mediating effect of this variable on the impact of negative gossip on employees’ organizational self-esteem, providing a basic reference for relevant organizational management of the world.

## Theoretical Analysis and Literature Review

### Negative Workplace Gossip and Organizational Self-Esteem

Self-esteem is defined as an individual’s overall assessment of his/her self-worth, reflecting the extent of an individual’s perception of “an individual who is capable and whose needs are met” (Korman, 1971). Organizational self-esteem reflects the degree to which employees believe they can fulfill their needs by playing a corresponding role in the organization (Pierce et al., 1989). In addition, it reflects whether individuals perceive their importance, value, and significance in the organization. Employees’ organizational self-esteem inextricably correlates with the environment in which they work. According to the self-consistency theory, employees’ self-concept is influenced by influential others in the organization. When people handle information, they selectively associate it with self-concept based on the influence of information on self-worth and self-perception.

In an organization, gossip is the main tool to fortify informal employee relations. Such “negative informal evaluative conversation in an organization about another member of the organization who is not present” is called negative workplace gossip (Mccaughey et al., 2013; Yue et al., 2015). The existing studies investigated the antecedents of negative workplace gossip, such as the value of individual factors, and the level of organization (Ellwardt et al., 2012) and organizational integrity, power structure, and so on. Some studies (Richman and Leary, 2009; Jose et al., 2015; Du and Zhu, 2018) claimed that negative workplace gossip is a type of damage to employees’ social relations, and it is challenging for employees who are surrounded by this negative gossip to trust others or establish a good cooperative relationship (Wu et al., 2018). Meanwhile, negative workplace gossip exerts a great negative impact on employees, such as decreasing employees’ work efficiency and job satisfaction. The disadvantages to their team far outweigh the benefits. In the existing research, few studies (Du and Zhu, 2020) investigated the negative workplace gossip from the perspective of the gossiped. In addition, we know very little about how negative workplace gossip affects work-related behavior, especially the process in which perceived negative workplace gossip might affect employees’ organizational self-esteem (Brady et al., 2017). According to the self-consistency theory, the perception of negative workplace gossip essentially depicts the belief that other members of the organization have a negative view of the person being gossiped, which indicates that negative workplace gossip might negatively affect employees’ organizational self-esteem. Hence, the following assumption is proposed:

*H1*: Negative workplace gossip exerts a significant negative effect on employees’ organizational self-esteem.

### The Intermediary Role of Workplace Exclusion

Workplace exclusion implies that employees deliberately ignore or crowd out other members in the workplace, including silent treatment, turning a blind eye and avoiding eye contact (Ferris et al., 2016). As a negative behavior, this phenomenon is standard in the workplace. It has the following crucial characteristics: first, workplace exclusion is a subjective feeling that employees can perceive, which has nothing to do with the objective existence of exclusion or the repeater’s motivation (Hitlan and Noel, 2009). Even if the colleagues around them are not deliberately isolated and excluded, as long as the employee subjectively feels overlooked or excluded, this constitutes workplace exclusion (Ferris et al., 2008). Second, workplace exclusion is characterized by the exclusion or neglect of individual employees, such as intentionally hiding useful information, disregarding treatment (Ferris et al., 2008). Third, the sources of workplace exclusion are complex and diverse, not only from within the team, including colleagues and bosses (Brady et al., 2017), but also from outside the team, even in the virtual world or when told by others to be excluded (Kong and Li, 2019). Workplace exclusion and negative workplace gossip are both forms of workplace bullying, but there are obvious differences and connections between them. Workplace bullying refers to a situation in which an employee is subjected to frequent and long-term negative behavior from a supervisor or colleague, and the imbalance between formal and informal power makes it difficult to resist and retaliate. The perpetrators of workplace bullying and workplace exclusion can come from different levels of the organization, and both have a negative impact on the object. When workplace exclusion causes persistent and repeated injury to the object, workplace exclusion becomes workplace bullying. Workplace exclusion and negative workplace gossip can both come from superiors and co-workers. However, different from workplace exclusion, the object of negative workplace gossip is not present (Yue et al., 2015). In addition, Ferris et al. (2008) pointed out that out-of-the-loop perceptions are subjective perceptions of external relationships. Then, when employees interpreted spiritual leadership behaviors in this context based on their perceived exclusion from leaders or colleagues. Workplace exclusion can make the excluded employees feel that they are not accepted by the organization, thus reducing their organizational identity, especially in the typical relations-oriented social context like China (Wu et al., 2010). As a standard phenomenon of “cold violence” in the workplace, although workplace exclusion is not as intense and direct as other negative behaviors, its negative impact is even worse (Wang et al., 2019), which not only seriously affects the individual’s psychological state, such as threatening the individual’s basic psychological needs and awakening the individual’s negative emotions (Jiang et al., 2011), but also exerts a negative impact on the individual’s work attitude. In addition, cold violence reduces individual work commitment and organizational commitment, and even reduces individual prosocial behavior, initiative behavior, and induces negative work behavior (Ferris et al., 2016; Wu et al., 2016b). Thus, whether such behavior is intentional or not, as long as individuals feel excluded, it causes destructive consequences to their physical and mental health and exerts a significant impact on their cognition, emotion, and behavior (Yang and Treadway, 2018).

Some studies (Smart and Leary, 2009; Zhao et al., 2016) claimed that negative workplace gossip is a type of social damage to employees, and employees surrounded by this negative gossip find it difficult to trust others or establish a cordial relationship, and is more likely to be ostracized by other organizational members (Ellwardt et al., 2012). Meanwhile, negative workplace gossip brings great side effects to employees, such as decreasing employees’ work efficiency, job satisfaction, and organizational self-esteem. According to the self-assessment perspective, people in an organization consider their values and roles as part of their self-concept, and comments from other colleagues are one of the external sources of information for self-assessment (Li and Ling, 2011). In contrast, employees who have a negative impact on negative workplace gossip tend to combine external negative assessment with their own self-evaluation, thereby perceiving exclusion from members, which, in turn, exerts a negative impact on behavior. Maslow’s hierarchy of needs theory (Maslow, 2007) divides human needs into physiological needs, safety needs, social needs, respect needs and self-actualization needs from low to high. At present, many scholars at home and abroad (Hu, 2015; Jin and Luo, 2019) based on Maslow’s theory of needs, made different exploration in enterprise management, education, cultural construction and other aspects, and believed that Maslow’s hierarchy of needs created on the basis of humanistic psychology had begun to integrate into all aspects of social life. Maslow’s hierarchy of needs theory highlights that the needs respected in social interaction are people’s high-level needs (Maslow, 2013), but workplace exclusion markedly suppresses people’s chances of acquiring these needs. When people feel isolated and excluded in the workplace, they tend to decrease mental effort to not spend more energy, thereby exhibiting negative interpersonal interaction behavior (Du and Zhu, 2020). As a result, workplace exclusion significantly reduces the excluded object’s sense of belonging, self-esteem, and even existence significance, especially when the excluded object does not understand the reasons for exclusion. In addition, a sense of belonging and self-esteem are more hurt. Pierce et al. (1989) claimed that organizational self-esteem is created by individuals in interpreting and perceiving the behavioral motivation and attitude of organizational members, and it reflects the matching of organizational cognition and self-cognition to some extent. Thus, it can be inferred that if individuals lack positive feedback from organizational members, the components of their negative self-cognition will increase accordingly, while the degree of organizational self-esteem would decline correspondingly. Meanwhile, some studies (Zhang, 2021) highlighted through meta-analysis that organizational context is a crucial predictor of organizational self-esteem, and signals transmitted by organizational environment or team members, such as organizational support and organizational identity, are a critical source of employees’ organizational self-esteem (Yu and Zhang, 2016). Workplace exclusion behavior is a type of serious negative feedback for individuals, which conveys negative signals from organizations on their cognition and evaluation; it makes employees feel that their value cannot satisfy the organization’s expectations. Thus, it gives a negative adjustment to the cognition of individual self-worth, and then decreases the individual’s perception of organizational self-esteem (Wu et al., 2010; Soltani et al., 2014; Zhao et al., 2020). Hence, we propose the following assumption:

*H2*: Workplace exclusion plays an intermediary role in the correlation between negative workplace gossip and organizational self-esteem.

### Differential Atmosphere Perception

Differential atmosphere refers to the difference in the relationship between near and far formed by an organization members centered on the person who holds organizational resources (Peng and Zhao, 2011). In China, the differential treatment in the differential atmosphere will results in different degrees of resource allocation for employees. Employees who perceive the atmosphere of organizational differentiation will have feelings of marginalization (Luo et al., 2016), thus affecting the psychology and behavior of employees. Therefore, it is of great significance to study the relationship between negative workplace gossip and organizational self-esteem from the perspective of organizational differential atmosphere. Differential atmosphere denotes the consensus level of organizational self-esteem. In Western culture, the leadership formed in the organization—the differentiation of member exchange relationships—is similar to the Chinese circle culture, but the differential atmosphere clearly shows the role of non-work relationships in the workplace (Shen et al., 2019; Kong et al., 2020). Managers in organizations are based on their loyalty and to treat and interact with employees with their intimate relationships. Employees in the organization exert a crucial impact on the perception of differential atmospheres in the workplace owing to these differential management behaviors, as well as exert a crucial impact on behavioral guidelines, thinking, and management operations in daily work. For instance, in the enterprise led by the Chinese, there exists a phenomenon that treats the subordinates, and they divide their employees into two people outside the circle, and a difference exists in resource allocation and emotional communication. This way, when the layer structure is formed, it is easy to create a dense or light workplace differential atmosphere (Han and Shen, 2018). The differential atmosphere in the workplace essentially depicts the distribution of resources and even power in the organization, which plays a vital role in organizational operation (Kong et al., 2020). For instance, an intense workplace differential atmosphere exerts a serious negative impact on the cohesiveness of the team and hinders its integration and collaboration to some extent, ultimately affecting the innovative ability of the organization and performance (Xue, 2017; Huang et al., 2018). “Circle” can often get more opportunities and resources in the workplace, as well as have higher upper and lower values match and loyalty to managers. “Landscapes” is perceived by the inclination of internal resource allocation, which is marginalized in tissues, which, in turn, leads to further enhancement of pressure (Lin et al., 2017).

In the environment of poor organizational order, employees’ perceived unfair treatment due to negative workplace gossip does not match their fair and just values (Redding, 1990; Liu et al., 2009), which is highly likely to cause employees’ cognitive dissonance. The sense of unfairness in the organization, such as resource tilt, interpersonal conflict, and emotional antagonism, further escalates stress and even in the workplace (Neubert et al., 2008). In organizations where the poor-order atmosphere is relatively weak, employees might enhance their perception of matching their superiors’ values because they perceive an atmosphere of fairness and justice (Chen et al., 2015). Meanwhile, the perception of these positive emotions is internalized into recognition and support from the organization, which positively enhances employees’ organizational self-esteem (Mcallister and Bigley, 2002). Quite the reverse, in organizations with a strong poor-order atmosphere, “insiders” and “outsiders” are easy to form a state of hostility because of the bias in the allocation of resources and the closeness of the interaction between superiors and subordinates (Wu and Peng, 2018). Owing to the “favor” of managers, “insiders” think that their power space is constantly expanding, which leads to identity deviation. According to the focus adjustment theory, employees who are “outsiders” or on the edge of the center of power lower the sense of security among colleagues because of biased treatment, resulting in a stronger sense of unfairness and stress. In turn, it negatively affects employee’s organizational self-esteem (Ferris et al., 2009; Ling and Cheng, 2017; Wang, 2020). Hence, the following is proposed:

*H3*: The perception of the differential-order atmosphere plays an intermediary role in the correlation between negative workplace gossip and organizational self-esteem.

**Figure 1:**
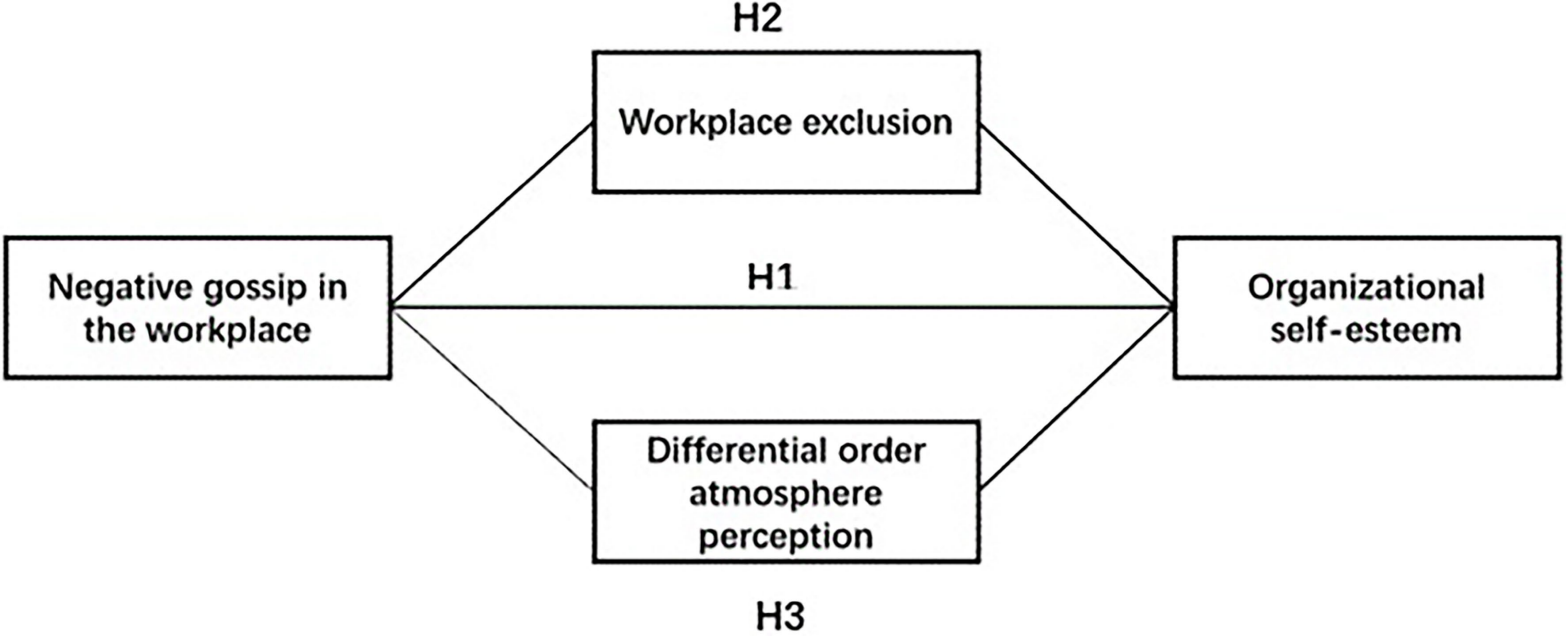
Theoretic analysis framework.

By summarizing the above theoretical analysis and research hypothesis, we can use the Figure 1 to generalize and describe the relationship between the core variables studied in this paper.

As can be seen from Figure 1, this paper mainly studies the impact of negative workplace gossip, workplace exclusion and the perception of the differential-order atmosphere on employees’ organizational self-esteem. Among them, negative workplace gossip has a direct negative impact on employees’ organizational self-esteem, which is also the core hypothesis of this paper. From the perspective of the possible influence path, on the one hand, negative workplace gossip influences employees’ organizational self-esteem by strengthening workplace exclusion; On the other hand, negative workplace gossip has an impact on employees’ organizational self-esteem by influencing their perception of the differential-order atmosphere.

## Research Methods

### Research Sample

The survey samples were selected from seven enterprises and institutions in Shandong, Shanghai, and Nanjing, including state-owned enterprises, institutions, and private enterprises. The industry involves automobile production, machinery manufacturing, software development, electronic communication, biotechnology, research institutes and planning and design. The total fixed assets of all units are more than 50 million yuan, and the number of employees is more than 800. All employees work 5 days a week. This paper adopts online and offline data collection methods. When collecting online data, we sent the link of the electronic questionnaire to the person in charge of the corresponding enterprise by email for the employees to fill in. The collected electronic questionnaire was saved in a special computer folder with the help of software tools. For offline data collection, we collected questionnaires at the enterprise site. After completing the questionnaire, the respondents sealed the paper questionnaire in an envelope as required and saved it in the questionnaire recycling box. Before the formal survey, to augment subjects’ understanding of the questionnaire as much as possible, a presurvey was conducted, and the questionnaire was revised and further improved per the feedback and suggestions of the members participating in the presurvey. To minimize the impact of common method bias on the research results, we collected data at three time-points at an interval of 1 month and measured the relevant demographic variables, including gender, age, education, position, length of service, and negative workplace gossip questionnaire, at the first time-point. At the second time-point, the measurement variables included workplace exclusion and differential atmosphere perception. At the third time-point, the measurement variable was employee organizational self-esteem. In the first survey, a total of 350 questionnaires were distributed, and 298 valid questionnaires were collected. One month later, 253 valid questionnaires were collected from the samples with contact information. After 1 month, the samples with contact information in the second survey were collected for the third time, and a total of 228 valid questionnaires were collected, with a recovery rate of 65.14%.

The public sector and private sector accounted for 25% and 75%, respectively, in the sample. Regarding gender, women accounted for 50% and men accounted for 50%. According to age, most survey samples aged <40 years, of which 25% aged <30 years, 30.3% aged 35 years, 30.3% aged 36 years, and 24.1% aged 40 years. Regarding education, high school (including vocational high school) and below accounted for 4.8%, junior college accounted for 22.8%, undergraduate accounted for 38.2%, graduate students accounted for 32.0%, and doctoral students accounted for 2.2%. Most survey samples were employees at the production line, accounting for 62.3%, and those who had worked for >10 years in the enterprises to which they belong were the most, accounting for 32.5%. The detailed structure of the samples is shown in Table 1.

**Table 1:** Structure of the samples (*N* = 228).

Category	Characteristic	Sample size		Percentage	
		Private sector	Public sector	Private sector	Public sector
Gender	Men	65	49	28.5%	21.5%
	Women	54	60	23.7%	26.3%
	≤25	18	14	7.9%	6.1%
Age	26–30	20	5	8.8%	2.2%
	31–35	58	11	25.4%	4.8%
	36–40	36	19	15.8%	8.3%
	41–45	26	2	11.4%	0.9%
	≥46	13	6	5.7%	2.6%
Qualifications	High school and below	9	2	3.9%	0.9%
	Junior college	28	24	12.3%	10.5%
	Undergraduate	70	17	30.7%	7.5%
	Master degree candidate	60	13	26.3%	5.7%
	Doctoral candidate	4	1	1.8%	0.4%
Job position	Employees who are at the production line	125	17	54.8%	7.5%
	Manager	46	40	20.2%	17.5%
Seniority	≤1 year	15	3	6.6%	1.3%
	1–3 years	18	13	7.9%	5.7%
	4–5 years	43	8	18.9%	3.5%
	6–10 years	32	22	14.0%	9.6%
	>10 years	63	11	27.6%	4.8%

### Variable Measurement

All the variable measurement scales in this study drew lessons from the maturity scales provided in previous studies (Pierce et al., 1989; Ferris et al., 2008; Chandra and Robinson, 2010; Wu et al., 2018); these scales have good reliability and validity in different research situations. Before the investigation, we followed the standard translation–back-translation procedure to ensure the accuracy and understandability of the questionnaire. All the questionnaire items were scored by Likert five-point, ranging from “very disagree” to “very agree.”

#### Negative Workplace Gossip

The measurement of this variable followed the measurement of Chandra and Robinson (2010), with a total of three questions, such as “other colleagues have made negative evaluations for me.” In this study, the Cronbach *α* of the meter was 0.928, suggesting that the scale has a good internal consistency.

#### Workplace Exclusion

The measurement of this variable used the measurement of Ferris et al. (2009), with a total of 10 questions, such as “In the past, my feelings or views will be ignored.” In this study, the Cronbach *α* of the meter was 0.960, suggesting that the scale has a good internal consistency.

#### Differential Atmosphere

The measurement of this variable followed Wang (2020), with a total of 11 questions, such as “The competent will share his ideas and practices in the team.” In this study, the Cronbach *α* of the meter was 0.934, suggesting that the scale has a good internal consistency.

#### Organize Self-Esteem

The measurement of this variable followed Pierce et al. (1989) development measures, with a total of 10 questions, such as “I think, I have a certain influence in the company.” “In this study, nine questions were selected, and the Cronbach *α* of the meter was 0.962, suggesting that the scale has a good internal consistency.

#### Control Variable

Considering the possible and reasonable impact on the results, we selected gender, age, academic qualifications, position, working age, and enterprise scale as control variables.

### Data Analysis Method

In this study, we used SPSS23.0 and MPLUS8 for statistical analysis. First, a relevant analysis was used for initial testing of hypotheses, followed by the structural equation model, where the verification factor analysis was used to test the model fitting, which, in turn, makes a hypothesis test.

## Results

### Common Method Deviation Inspection

To control the common method deviation, we used the Harman single-factor test method, and the first main component obtained at the time of rotation was 43.659%, which was lower than the critical point recommended by Hair et al. (2011). Thus, the common deviation had a limited effect in this study.

### Verification Factor Analysis

To ensure that all variables in the model had better distinction, we compared the model using the confirmatory factor analysis (CFA; Table 2). Table 2 shows that the four-factor model fits better (*χ*^2^ = 896.622, df = 489, TLI = 0.937, CFI = 0.942, RMSEA = 0.060, SRMR = 0.051) and is superior to other models.

**Table 2:** Confirmatory factor analysis and comparison (*N* = 228).

Model	*χ* ^2^	df	TLI	CFI	RMSEA	SRMR
Four-factor model	896.622	489	0.937	0.942	0.060	0.051
(A, B, C, D)						
Three-factor model 1	2321.767	492	0.719	0.738	0.128	0.173
(A, B + C, D)						
Three-factor model 2	1653.404	492	0.822	0.834	0.102	0.190
(A + C, B, D)						
Three-factor model 3	1662.316	492	0.820	0.833	0.102	0.077
(A, C, B + D)						
Two-factor model	2393.373	494	0.709	0.728	0.130	0.198
(A + C, B + D)						
Single-factor model	3316.249	495	0.569	0.596	0.158	0.183
(A + B + C + D)						

A, negative workplace gossip; B, workplace exclusion; C, differential atmosphere perception; and D, employee organizational self-esteem.

### Descriptive Statistical Analysis

Table 3 shows the mean value, standard deviation of each research variable, and the correlation coefficient between variables. Mean and standard deviation, respectively, represent the general level and dispersion degree of each variable data, while correlation coefficient can reflect the linear correlation between the two variables. The mean value and standard deviation of the nine variables are all within a reasonable range, which also provide basic conditions for subsequent hypothesis testing. From the correlation coefficient, negative gossip in the workplace is significantly negatively correlated with organizational self-esteem of employees (r = −0.64, *p* < 0.01), which is consistent with H1 proposed above. A significant positive correlation was found between negative workplace gossip and workplace exclusion (*r* = 0.81, *p* < 0.01). In addition, a significant negative correlation exists between workplace exclusion and employee organizational self-esteem (*r* = −0.75, *p* < 0.01). A positive correlation exists between negative gossip and differential-order atmosphere perception (*r* = 0.20, *p* < 0.01). These correlation coefficients aligned with the theoretically expected relationship and provide preliminary indirect evidence for the mediation mechanism examination in the next section.

**Table 3:** Descriptive statistics and related analysis results (*N* = 228).

Variable	Mean	Standard deviation	1	2	3	4	5	6	7	8	9
1. Gender	1.50	0.50									
2. Age	3.38	1.50	−0.05								
3. Education	3.04	0.91	−0.01	0.05							
4. Position	1.65	0.94	−0.16^*^	0.10	0.38^**^						
5. Working experience	3.59	1.28	−0.07	0.51^**^	0.36^**^	0.40^**^					
6. Negative workplace gossip	2.61	1.31	−0.04	0.09	−0.05	−0.12	−0.04	**0.90**			
7. Differential atmosphere perception	3.61	0.90	−0.03	0.50^**^	0.00	−0.04	0.30^**^	0.20^**^	**0.75**		
8. Workplace exclusion	2.57	1.12	−0.08	0.05	−0.16^*^	−0.22^**^	−0.08	0.51^**^	0.18^**^	**0.84**	
9. Employee Organizational Self-esteem	3.37	1.10	−0.03	0.06	0.17^**^	0.32^**^	0.14^*^	−0.64^**^	−0.01	−0.75^**^	**0.83**

The value in bold on the diagonal is the square root of each variable AVE.

** indicate *p* < 0.01;

* indicate *p* < 0.05.

In addition, the relationship between negative gossip and employee’s personal factors (gender, age, education level, and position level) also shows different characteristics. First, age is positively correlated with negative gossip, indicating that the attitude of employees toward gossip will change with the increase of age, and the older they are, the stronger the correlation with negative gossip is. Second, gender is negatively correlated with negative gossip. The possible reason is that female employees in organizations are more vulnerable to negative gossip in the workplace influenced by gender bias in Chinese traditional culture. Thirdly, education level is negatively correlated with negative gossip, showing that the higher the education level is, the less likely the employees are to be attacked by negative gossip, while those employees with lower education level are vulnerable in the organization and are also vulnerable to the attack of negative gossip. Fourth, position level is negatively correlated with negative gossip, illustrating that individuals with weak power status in organizations are more vulnerable to negative gossip attacks due to lack of social support.

### Hypothesis Test Results

#### Test of Relationship Between Variables Based on Hierarchical Regression

Using hierarchical regression analysis, we explored the internal relationships between negative workplace gossip, perception of differential atmosphere, workplace exclusion, and employee organizational self-esteem under different models (Table 4).

**Table 4:** Hierarchical regression results.

Variable	Employee organizational self-esteem			Workplace exclusion		Differential atmosphere perception	
	Model 1	Model 2	Model 3	Model 4	Model 5	Model 6	Model 7
Gender	0.059	−0.020	−0.109	−0.245	−0.142	−0.066	−0.050
Age	0.015	0.076	0.012	0.059	−0.020	0.269	0.257
Education	0.048	0.061	−0.028	−0.100	−0.117	0.016	0.013
Position	0.353	0.269	0.195	−0.250	−0.140	−0.142	−0.124
Length of service	−0.016	−0.050	−0.041	−0.008	0.036	0.108	0.115
Enterprise size	0.067	0.051	0.074	−0.013	0.009	−0.090	−0.087
Negative workplace gossip		−0.517^***^			0.374^***^		0.105^**^
Workplace exclusion			−0.732^***^				
Differential atmosphere perception			0.173^**^				
R^2^	0.111	0.478	0.608	0.074	0.679	0.285	0.398
Adj-*R*^2^	0.087	0.462	0.594	0.049	0.669	0.266	0.386
*F*-value	4.593^**^	28.815^**^	42.548^**^	2.929^*^	66.622^**^	14.689^**^	23.968^**^

*** indicate *p* < 0.001.

** indicate *p* < 0.01.

* indicate *p* < 0.05.

Model 1 in Table 4 is a benchmark model to validate the correlation between control variables and employee organizational self-esteem. Model 2 adds the variable of negative workplace gossip based on model 1. Table 4 shows that its explanatory power is significantly improved: Adj-*R*^2^ increased from 0.087 to 0.462, and the *F*-value was 28.815 (*p* < 0.001). Thus, negative workplace gossip adversely affects employee organizational self-esteem, and H1 is established.

Model 3 is based on the benchmark model 1, and validates the correlation between workplace exclusion, perception of difference atmosphere, and employee organizational self-esteem. In addition, adj-*R*^2^ increased from 0.087 to 0.594, and the *F*-value was 42.548 (*p* < 0.001). Thus, workplace exclusion negatively affects employees’ organizational self-esteem, and the perception of differential atmosphere positively affects employees’ organizational self-esteem.

Model 4 is a benchmark model of control variables and mediation variables for workplace exclusion, confirming the correlation between control variables and workplace exclusion. Model 5 adds variable negative workplace gossip based on the benchmark model 4 to validate the correlation between negative workplace gossip and workplace exclusion. Compared with model 4, the explanatory ability of model 5 improved significantly: Adj-*R*^2^ increased from 0.049 to 0.669, and the *F*-value was 66.622 (*p* < 0.001). Thus, negative workplace gossip positively affects workplace exclusion.

Model 6 is a benchmark model of differential atmosphere perception between control variables and mediator variables, validating the correlation between control variables and differential atmosphere perception. Model 7 adds variable negative workplace gossip based on the benchmark model 6, verifying the correlation between negative workplace gossip and perception of differential-order atmosphere. Compared with model 6, the explanatory ability of model 7 improved significantly: Adj-*R*^2^ increased from 0.266 to 0.286, and the *F*-value was 23.968 (*p* < 0.001). Thus, negative workplace gossip positively affects the perception of a differential atmosphere.

#### The Mediating Role of Workplace Exclusion and Perception of Differential Atmosphere

The study in the previous section shows that both workplace exclusion and differential-order atmosphere perception have a mediating effect on the relationship between workplace negative gossip and organizational self-esteem, but the extent and type of the mediating effect cannot be clearly defined. Therefore, we continue to analyze the mediating effect of workplace exclusion and differential-order atmosphere perception by using Bootstrap method. The Bootstrap method (Hayes, 2009) was used in this study because its test results are more reliable and it is generally recognized and used by scholars. Thus, Bootstrap re-sampling analysis was done of 228 questionnaire sample data 5,000 times, and the results are shown in Table 5.

**Table 5:** Bootstrap analysis results of mediation effect.

Action path	Direct effect			Mediation effect		
	95% confidence interval			95% confidence interval		
	Effect size	Lower limit	Upper limit	Effect size	Lower limit	Upper limit
Negative workplace gossip→workplace exclusion→employee organizational self-esteem	−0.108	−0.230	0.014	−0.410	−0.530	−0.296
Negative workplace gossip→differential atmosphere perception→employee organizational self-esteem	−0.535	−0.617	−0.452	0.017	0.001	0.042
Negative workplace gossip→workplace exclusion, perception of bad order atmosphere→employee organizational self-esteem	0.122	−0.243	−0.002	−0.395	−0.515	−0.280

First, we examined the mediating role of workplace exclusion. The test results revealed that the impact of workplace exclusion between negative workplace gossip and employee organizational self-esteem was −0.410, with a 95% confidence interval (CI) of [−0.530, −0.296], suggesting that workplace exclusion exerts a significant mediating effect. After adding the variable of workplace exclusion, the direct effect of negative workplace gossip on employees’ organizational self-esteem was −0.108, and 95% CI was [−0.230, 0.014], including 0, indicating that differential atmosphere perception plays a completely mediating role. Hence, H2 is established.

Second, we examined the mediating role of differential-order atmosphere perception. The impact of differential-order atmosphere perception between negative workplace gossip and employees’ organizational self-esteem was 0.017, and 95% CI was [0.001, 0.042], suggesting that differential-order atmosphere perception exerts a significant mediating effect. After adding the variable sequence atmosphere perception, the direct impact of negative workplace gossip on employee organizational self-esteem was −0.535, and 95% CI was [−0.617, −0.452], excluding 0, suggesting that differential sequence atmosphere perception plays a part in the mediating role. Hence, H3 is established.

Finally, we examined the dual mediating role of workplace exclusion and perception of differential atmosphere. Table 5 shows that the direct impact of negative workplace gossip on employees’ organizational self-esteem was 0.122 after adding the double mediation variables, and 95% CI was [−0.243, −0.002], excluding 0; the total effect of double mediation was–0.395, and 95% CI was [−0.515, −0.280], excluding 0, suggesting that workplace exclusion and perception of differential atmosphere can play a dual mediating role. Among them, the mediating effect value of workplace exclusion was −0.414, the direct effect value of differential atmosphere perception was 0.019, and 95% CI was [−0.532, −0.302] and [0.004, 0.042], respectively, none of which contained 0.

## Discussion

From the viewpoint of negative workplace gossip and with the help of self-consistency theory, this study investigated the mechanism and boundary conditions of negative workplace gossip affecting employees’ organizational self-esteem and constructed a multiple intermediary model. Through the three-stage survey data of a sample of 228 members of 7 teams, we found that negative workplace gossip exerts a negative impact on employees’ organizational self-esteem. Workplace exclusion plays a complete intermediary role between negative workplace gossip and employees’ organizational self-esteem, and the perception of poor-order atmosphere plays a partial intermediary role.

The theoretical contribution of this study is primarily reflected in the following aspects: first, it expands the relevant literature on the influencing factors of organizational self-esteem. In the previous literature, many studies examined the role of variables such as employee self-efficacy (Pierce et al., 1989; Bowling and Eschleman, 2010), employee optimism (Lee, 2003), psychological resilience (Dumont and Provost, 1999) in affecting employees’ organizational self-esteem from the standpoint of personal and organizational characteristics. Nevertheless, relatively few studies have been conducted on how negative workplace gossip affects employees’ organizational self-esteem. Existing study (Yue et al., 2015) only theoretically analyzed the direct negative impact of negative workplace gossip on organizational self-esteem, and lacked empirical studies on its influencing relationship and internal mechanism. This study enriches the relevant research on the influencing factors of employees’ organizational self-esteem by examining the mechanism and boundary conditions of negative workplace gossip affecting employees’ organizational self-esteem. In addition, the results of this study (Table 4) demonstrate that negative workplace gossip is not conducive to the establishment of organizational self-esteem of employees, and as a negative human–computer interaction experience, negative workplace gossip is a crucial factor affecting employees’ organizational self-esteem, which expands the explanatory boundary of the study that affects employees’ organizational self-esteem. Second, it reveals the influence mechanism of negative workplace gossip on employees’ organizational self-esteem. Some studies demonstrated that negative workplace gossip can affect employees’ organizational self-esteem by affecting organizational atmosphere, workplace exclusion, employees’ psychological empowerment, and employees’ organizational identity; this also provides some ideas for this study. Our findings (Table 5) revealed that negative workplace gossip could significantly augment workplace exclusion in organizations, and then negatively affect employees’ organizational self-esteem. Through relatively covert attacks, negative workplace gossip poses a serious threat to the mental and psychological resources of the gossiped, which makes them gradually rejected by most employees. The excluded employees are in a work atmosphere where they are not trusted and their work results are not valued, and lack of interpersonal connections with colleagues. As a result, work is difficult to meet their sense of belonging and the sense of value they experience and other needs, so that their organizational self-esteem is seriously hurt. Some scholars (Williams, 2007; Scott et al., 2013) came to the similar conclusions. This conclusion is a useful supplement and expansion of the existing research. Third, starting from the Chinese cultural context, this study examined the intermediary effect of the unique perception of differential-order atmosphere in Chinese culture. The research result (Table 5) reveals that the perception of differential-order atmosphere in the organization partly mediates the correlation between negative workplace gossip and employees’ organizational self-esteem. Negative gossip in the workplace can damage employees’ physical and mental health and bring negative emotions (Yue et al., 2015; Du and Zhu, 2018) and enhance the perception of differential atmosphere to some extent. Employees will receive negative signals about injustice and feel less support from the organization and supervisor, forming negative ego in the workplace (Yu and Zhang, 2016), thus reducing organizational self-esteem. This discovery further enhances the explanatory power of the existing research framework to employees’ organizational self-esteem.

In addition, the results in this paper verifies the negative effects of workplace negative gossip, but it is not consistent with the conclusions of some foreign scholars. For example, some scholars (Feinberg et al., 2014; Wu et al., 2016a) showed that employees who experienced workplace negative gossip would become more generous, show more cooperative behaviors and increase their contributions to the organization, and enhance the organizational self-esteem finally. The possible reason for these different conclusions above lies in the different effects caused by the cultural differences between China and the West. Influenced by Chinese traditional culture, the workplace negative gossip as a form of informal communication has become a natural part of Chinese enterprises, negative workplace gossip through a negative message by gossip is obstructive pressure source, and its damage to the organization atmosphere and interpersonal relationship, resulting in a loss of the employee organization self-esteem. The individual-centered differential pattern is the basic form of interpersonal communication in Chinese traditional culture, leading to the differential management atmosphere plays a negative role in the relationship between workplace negative gossip and organizational self-esteem.

The management enlightenment of this study primarily lies in the following. First, enterprises should focus on avoiding the negative impact of negative workplace gossip. In this study, negative workplace gossip was used as an independent variable to guide managers to comprehend the potential negative effects of negative workplace gossip from the employees’ viewpoint. Besides, enterprises should introduce clear rules and regulations, fortify the personal management of employees, and minimize negative workplace gossip. Conversely, enterprises should strengthen the emotional construction between employees, establish a good communication mechanism, and strive to control the breeding of negative workplace gossip from the source. Once negative effects emerge, managers should intervene in time to avoid further harm. For employees who are attacked by negative gossip, managers should pay timely and active attention to the emotional status of the gossips, and supplement the resources and energy of the employees by means of in-depth communication, personalized care and team building activities.

Second, considering the intermediary role of workplace exclusion between negative workplace gossip and employee organizational self-esteem, enterprises should focus on the ostracism among employees. Workplace exclusion behavior will not only create job problems for employees but also markedly hurt employees’ organizational self-esteem. Thus, corresponding measures must be taken to intervene reasonably. Moreover, organizational managers can prevent workplace exclusion by guiding employees to engage in fair competition. In addition, they should also try to avoid hiring employees with workplace exclusion tendencies. In contrast, when workplace exclusion occurs, managers should take positive protection and intervention measures to build a harmonious working environment and reinforce communication to alleviate the mental pain of excluded employees.

Third, this study demonstrates that negative workplace gossip is universal and difficult to trace. The emergence of this phenomenon closely correlates with the organizational atmosphere, and the strong differential atmosphere nourishes the emergence of negative workplace gossip, especially in the special context of “circle” culture. Thus, it is suggested that organizations should develop and maintain effective channels of information exchange and create a healthy, positive, and fair organizational culture. Essentially, organizations should fundamentally curb the emergence of workplace gossip. In addition, the existence space of differential atmosphere can be compressed through task arrangement and culture shaping. Improve the overall design of the organization and team work, clarify the task interface on the production value chain of the team, make the team members become an important link of value-added value, and guide the staff to complete the organizational tasks and innovation direction.

Fourth, pay attention to the different effects of negative gossip on organizational self-esteem caused by the differences between Chinese and Western cultures. Take corresponding management measures according to the internal reality of the enterprise. In the organization which oriental culture atmosphere is relatively strong, managers should pay attention to restrain the negative gossip in the workplace, at the same time, through the development object of organizational trust and fair management measures of organization atmosphere of difference expansion, maintain an atmosphere of fairness and justice within the organization.

## Conclusion and Future Research Direction

### Conclusion

Negative gossip is prevalent in the workplace and is a special and important way of interpersonal communication within organizations (Dunbar, 2004). Based on the self-consistency theory, this study explored the impact of negative workplace gossip on employees’ organizational self-esteem by constructing a mediating model and using 228 employee survey data. The findings are as follows: First, negative workplace gossip has a significant negative impact on employees’ organizational self-esteem. Second, workplace exclusion was a complete mediator between negative workplace gossip and organizational self-esteem. Third, the perception of differential atmosphere plays a partially mediating role in the relationship between employees’ organizational self-esteem and negative workplace gossip. On the one hand, this study helps to open the black box of the influence mechanism of negative gossip in the workplace, and provides theoretical support for further exploring the mechanism of the generation of organizational self-esteem in the context of negative cultural atmosphere. On the other hand, it is helpful for managers to properly intervene and manage negative gossip in the workplace, so as to improve the innovative performance of employees.

### Limitations and Future Research Direction

Some limitations of this study and future research areas are as follows:

First, the study has adopted mature and recognized negative workplace gossip, and the scale of China’s cultural context remains in the exploration stage; however, there is a lack of clear classification standards considering the impact of the differences between Chinese and Western cultural differences. Thus, future research could develop a negative gossip table with higher reliability and a valid workplace in the context of China. Moreover, all measurements were self-reported in this study, raising the risk of general methods.

Second, the nature and size of the sample also limited universal research. We assume that some control variables, including education, position, and working age, would affect, but the results showed no significant correlation between them, which could be owing to regional and scale restrictions. Besides, our study sample came from Shandong Province and Shanghai. Thus, future research could increase sample quantity and regional scope through cross-cultural research and choose different types of enterprises and employees.

Third, this study explored the mediation of workplace exclusion and differential atmospheres from the standpoint of self-consistency theory; however, there could be other potential intermediaries in this process. Regarding research design, other variables from employees or leadership could affect the organization of employees (such as psychological authorization, leadership membership, and leadership authorization). In the future, based on the alternative explanations of these variables, the conclusion of this study can be further confirmed.

## Data Availability Statement

The raw data supporting the conclusions of this article will be made available by the authors, without undue reservation.

## Ethics Statement

Ethical review and approval was not required for the study on human participants in accordance with the local legislation and institutional requirements. Written informed consent from the patients/participants was not required to participate in this study in accordance with the national legislation and the institutional requirements.

## Author Contributions

All authors contributed to the design and implementation of the research, the analysis of the results, and the writing of the manuscript and contributed to the article and approved the submitted version.

## Funding

This research was funded by Social Science Planning Project of Shandong Province in China (grant no. 21CRCJ03) and Jinan Philosophy and Social Science Project in 2021 of China (grant no. JNSK21C73).

## Conflict of Interest

The authors declare that the research was conducted in the absence of any commercial or financial relationships that could be construed as a potential conflict of interest.

## Publisher’s Note

All claims expressed in this article are solely those of the authors and do not necessarily represent those of their affiliated organizations, or those of the publisher, the editors and the reviewers. Any product that may be evaluated in this article, or claim that may be made by its manufacturer, is not guaranteed or endorsed by the publisher.
